# Response speed control of helicity inversion based on a “regulatory enzyme”-like strategy

**DOI:** 10.1038/s41598-017-16503-1

**Published:** 2018-01-09

**Authors:** Shiho Sairenji, Shigehisa Akine, Tatsuya Nabeshima

**Affiliations:** 1 Faculty of Pure and Applied Sciences, University of Tsukuba, 1-1-1 Tennodai, Tsukuba, Ibaraki, 305-8571 Japan; 2 Graduate School of Natural Science and Technology / Nano Life Science Institute (WPI-NanoLSI), Kanazawa University, Kakuma-machi, Kanazawa, 920-1192 Japan

## Abstract

In biological systems, there are many signal transduction cascades in which a chemical signal is transferred as a series of chemical events. Such successive reaction systems are advantageous because the efficiency of the functions can be finely controlled by *regulatory enzymes* at an earlier stage. However, most of artificial responsive molecules developed so far rely on single-step conversion, whose response speeds have been difficult to be controlled by external stimuli. In this context, developing artificial conversion systems that have a regulation step similar to the *regulatory enzymes* has been anticipated. Here we report a novel artificial two-step structural conversion system in which the response speed can be controlled based on a *regulatory enzyme*-like strategy. In this system, addition of fluoride ion caused desilylation of the siloxycarboxylate ion attached to a helical complex, resulting in the subsequent helicity inversion. The response speeds of the helicity inversion depended on the reactivity of the siloxycarboxylate ions; when a less-reactive siloxycarboxylate ion was used, the helicity inversion rate was governed by the desilylation rate. This is the first artificial responsive molecule in which the overall response speed can be controlled at the regulation step separated from the function step.

## Introduction

**Figure 1:**
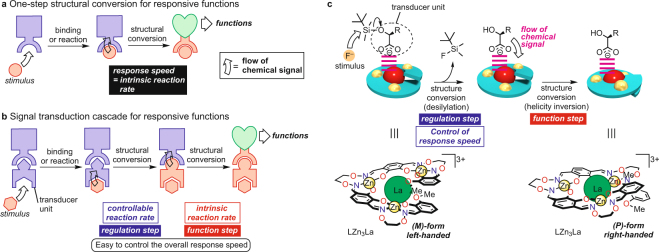
Concept and design of responsive functional systems based on a *regulatory* *enzyme*-like strategy. (**a**) One-step structural conversion for responsive function. (**b**) Multi-step structural conversion for responsive functions. The function activity (reaction rates) may be controlled at an earlier step called the regulation step. (**c**) Design of a new artificial system for helicity inversion mediated by desilylation of the coordinating siloxycarboxylate ions at the regulation step.

In responsive molecules using a chemical stimulus, binding with a chemical species causes a structural change that leads to responsive functions (Fig. 1a). Representative examples in biological systems are allosteric enzymes^1,2^, which undergo a structural change upon binding with an *effector*, resulting in a responsive function. There are also many artificial responsive molecules using chemical species as the trigger^3–8^, and some of them are used to drive molecular machines^9–11^. In these systems, the response speeds are determined by the intrinsic reaction rates of the structural conversion, which are usually difficult to change without changing the reaction conditions.

In biological systems, there are cascade systems in which a chemical signal is transferred as a series of chemical events prior to the structural changes leading to their functions (Fig. 1b)^12–21^. A significant feature of such successive reactions is that they have a *regulatory enzyme* (or a *rate-limiting enzyme*) that controls the efficiency of the functions not at the final function step, but at an earlier stage. This preceding step is important for fine-tuning of the overall activity. In artificial functional systems, however, there are rare examples of such signal transduction cascades whose functions are controlled at a prior stage in a series of two or more successive chemical events. Nevertheless, such a cascade system is advantageous, because a regulation step, which could control the overall response speed and/or time profiles of the functions, can be separated from the final function step (Fig. 1b). This would enable not only to switch on and off the functions, but also to set the activity at any level. In addition, the unique time-programmable features would be introduced in discrete functional molecular systems; such a time-programmable material, which has recently attracted increasing attention, has been achieved only in supramolecular aggregate systems^22^. In this context, developing artificial conversion systems that have a regulation step similar to the *regulatory enzymes* found in biomolecules has been anticipated.

Thus, we designed a novel simplified artificial system for a signal transduction cascade that enables a two-step conversion using fluoride ion as the signal input. The fluoride ion causes desilylation of a chiral siloxycarboxylate ion during the first step and this conversion controls the response speeds of the helicity inversion of dynamic helical complex LZn_3_La^23–25^ during the final step (Fig. 1c). Helicity inversion is one of the basic and important structural conversions^26–34^, because helical structures^35–39^ are ubiquitous structural motifs in various types of substances. In the present LZn_3_La system, the helicity is sensitively affected by structural differences in the chiral carboxylate ions^40,41^, whereas the helicity inversion rate is not significantly affected (thus called the *intrinsic helix inversion rate*, hereafter). These facts inspired us to design a system in which helicity inversion is driven by a *slow* chemical transformation in the coordinating carboxylate ions. In fact, there have been several helical metal complexes that can change their helix inversion rates^23,42,43^ by replacing the central metal ion. The time-programming in these systems needs to change the *intrinsic helix inversion rates*, whereas the helix inversion rates of the present system can be controlled at the regulation step without changing the *intrinsic helix inversion rates*. We now report this new type of two-step structural conversion in which the response speed of the helicity inversion at the final function step was effectively controlled at the regulation step using siloxycarboxylate ions with different reactivities.

## Results and Discussion

**Figure 2:**
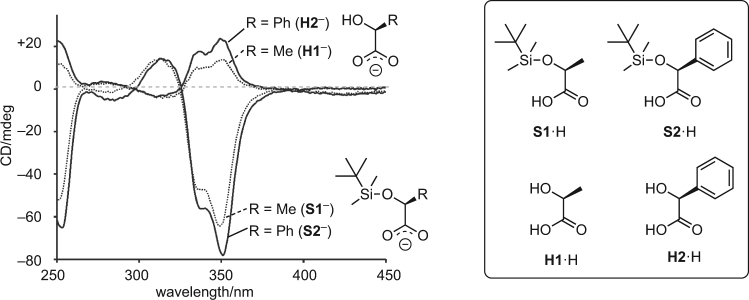
CD spectra of LZn_3_La (0.20 mM, acetonitrile/chloroform, 9:1, path length 1 mm, 295 K) in the presence of 3 equiv of chiral carboxylic acids (**S1·**H, **S2·**H, **H1·**H, and **H2·**H) and 3 equiv of DABCO.

Requirements for the F^−^-triggered helicity inversion in this system is that the carboxylate ions before and after the desilylation should induce opposite helicities of the LZn_3_La. Thus, we investigated the CD spectra of LZn_3_La in the presence of several chiral carboxylic acids (**S1**·H, **S2**·H, **H1**·H, and **H2**·H) (Fig. 2). DABCO (1,4-diazabicyclo[2.2.2]octane) was used to deprotonate these carboxylic acids. We have already demonstrated that chiral carboxylate ions such as **H1** ^−^ and **H2** ^−^ efficiently shift the *P*/*M* equilibrium of the LZn_3_La helix and that two molecules of these carboxylate ions can interact with LZn_3_La from the CD spectroscopic titration experiments^40,41^. When the siloxycarboxylate ion, **S1** ^−^ or **S2** ^−^, was present, a negative Cotton effect was observed at 350 nm, which is indicative of the (*M*)-helicity of LZn_3_La based on a comparison with related complexes^44–47^. In contrast, a positive Cotton effect was observed at 350 nm when the hydroxycarboxylate ion, **H1** ^−^ or **H2** ^−^, was present under the same conditions. The observed differences in the signs of the Cotton effect should be attributed to the opposite preference of the (*M*)- and (*P*)-forms. Consequently, the-hydroxycarboxylate ions and the corresponding siloxy derivatives induced opposite helicities although they have the same stereoconfiguration. Therefore, we expected that, if the silyl group in **S1** ^−^ and **S2** ^−^ is removed by the reaction with fluoride ion, a responsive helicity inversion should take place.

**Figure 3:**
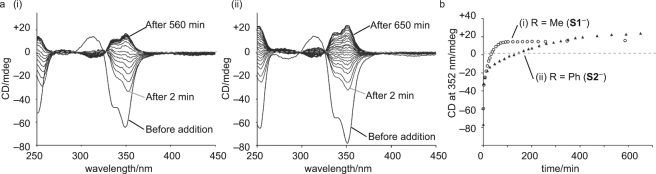
CD spectral observation of helicity inversion triggered by F^–^ addition. (**a**) CD spectral changes of LZn_3_La (0.20 mM, acetonitrile/chloroform, 9:1, path length 1 mm, 295 K) in the presence of siloxycarboxylic acid (3 equiv) and DABCO (3 equiv) after the addition of tetrabutylammonium fluoride (i, 3 equiv for **S1·**H; ii, 4 equiv for **S2·**H). (**b**) Time course of the CD intensity changes (352 nm).

Indeed, the addition of fluoride ion caused significant changes in the CD spectra. While the siloxycarboxylate ion **S1** ^−^ induced a negative Cotton effect at 350 nm attributable to the (*M*)-helicity of LZn_3_La (Fig. 3a,i), the Cotton effect started to immediately decrease after the addition of 3 equiv of fluoride ion. The intensity decreased with approximate first-order kinetics and turned positive after 30 min. The spectral changes were almost completed after 100 min (Fig. 3 b,i) to result in a CD spectrum similar to that of the (*P*)-helical LZn_3_La in the presence of **H1**·H and DABCO (Fig. 2). This suggested that the siloxycarboxylate ion **S1** ^−^ coordinating to LZn_3_La was converted into the desilylated derivative **H1** ^−^. This was clearly evidenced by the ESI-MS peak (*m*/*z* = 611.0 for [LZn_3_La + **H1**]^2+^) observed in the solution after reaction with the fluoride ion (Supplementary Fig. S3).

Interestingly, the structures of the siloxycarboxylate ions significantly affected the response speeds of the helicity inversion. We similarly prepared the (*M*)-helical LZn_3_La complex by using the mandelate-based siloxycarboxylate ion **S2** ^−^ in place of the lactate-based **S1** ^−^. This helical complex, LZn_3_La with **S2** ^−^, also showed a gradual decrease in the CD intensity after the addition of 3 equiv of fluoride ion, but the reaction was so slow that the CD signal did not turn positive even after 720 min (Supplementary Fig. S5). When the amount of fluoride ion was increased from 3 equiv to 4 equiv (Fig. 3a,ii), the helicity was inverted as observed for LZn_3_La with **S1** ^−^. However, the reaction was still significantly slow compared to the LZn_3_La–**S1** ^−^ system; the CD signal turned positive after 120 min, but it took 650 min to complete the reaction (Fig. 3b,ii). The resultant CD spectrum ( + 23.9 mdeg at 350 nm, Fig. 3a,ii) was very similar to that of LZn_3_La in the presence of **H2**·H and DABCO ( + 23.7 mdeg at 350 nm, Fig. 2). This indicated that the siloxycarboxylate ion **S2** ^−^ coordinating to LZn_3_La underwent desilylation to give the hydroxycarboxylate **H2** ^−^. This was confirmed by the ESI-MS peak after the reaction (*m/z* 641.9 for [LZn_3_La + **H2**]^2+^, Supplementary Fig. S6).

**Figure 4:**
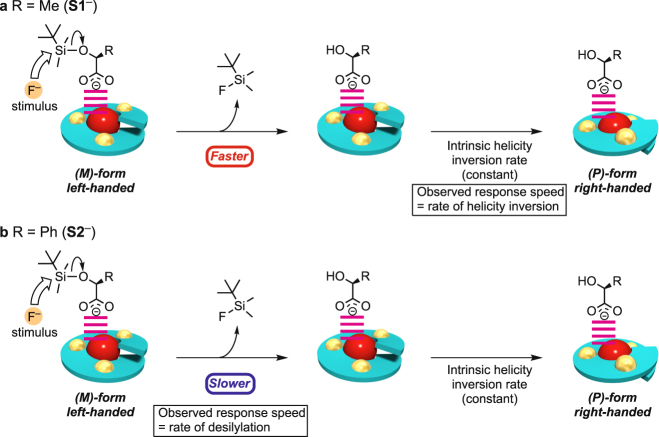
Schematic drawing for the helicity inversion triggered by F^–^ addition. (**a**) R = Me (**S1** ^−^). The desilylation rate is very fast and the overall helicity inversion rate is governed by the *intrinsic helicity inversion rate*. (**b**) R = Ph (**S2** ^−^). The desilylation rate is slower than the *intrinsic helicity inversion* *rate* and governs the overall helicity inversion rate.

As already described, it is clear that the helicity inversion of LZn_3_La was triggered by the fluoride ion via the desilylation of **S1** ^−^ or **S2** ^−^ coordinating to the LZn_3_La helical complex. However, the LZn_3_La–**S1** ^−^ system showed significantly faster response than the LZn_3_La–**S2** ^−^ system. This difference should mainly arise from the different reactivity of the silyl groups in the carboxylate ions **S1** ^−^ and **S2** ^−^ toward the fluoride ion. In the case of the lactate-based **S1** ^−^, the silyl group was completely removed within 3 min (Supplementary Fig. S4), which was evidenced by the ^1^H NMR analysis. Since the observed half-life of the CD intensity changes (*t*_1/2_ ≈ 20 min) was much longer than that of the desilylation (*t*_1/2_ < 1 min), the response speed of the helicity inversion should be governed by the *intrinsic helix inversion rate* of the LZn_3_La scaffold^44,45^ (Fig. 4a). On the other hand, the ^1^H NMR analysis indicated that the desilylation of **S2** ^**−**^ was very slow; the unreacted **S2** ^**−**^ still remained even after 120 min (Supplementary Fig. S7). It should be noted that the observed response speed of the helicity inversion is much slower than the *intrinsic helix inversion rate* of the LZn_3_La scaffold. Obviously, the observable overall response speed of the helicity inversion is controlled at the desilylation step (Fig. 4b). Therefore, the helicity inversion of LZn_3_La was triggered by the fluoride ion, and the response speed was controlled at the regulation step of the signaling cascade by using the siloxycarboxylate ion without changing the *intrinsic helix inversion rate*.

In summary, we have developed a new artificial signal transduction cascade system for controlling the helicity inversion speeds. The fluoride ion triggered two successive chemical events, e.g., desilylation of the siloxycarboxylate ions followed by helicity inversion of the LZn_3_La dynamic helix. The overall response speed was efficiently controlled at the regulation step of the signaling cascade, just like *regulatory enzymes* in biological systems, by using the slower desilylation of the siloxycarboxylate ions without changing the *intrinsic helix inversion rates*. Before this study, the control of the response speeds of functional molecules had been believed to require modification of their parent molecular framework. Our research of the function tuning at the regulation step in a signal transduction cascade could be applied to a variety of functional molecular systems that can control the response speed without altering the intrinsic nature of the functional molecules. In addition, this fine-tuning of the response speeds would open the way to new chemistry in which molecular machinery motions and chemical functions are controlled in a time-programmable fashion.

## Methods

### General procedures

All chemicals were reagent grade and used without further purification. Column chromatography was performed with Kanto Chemical silica gel 60 N (spherical, neutral). ^1^H NMR spectra were recorded on a Bruker AVANCE600 spectrometer (600 MHz), a Bruker DPX400 (400 MHz), or a Bruker AVANCE400 spectrometer (400 MHz). In NMR measurements, tetramethylsilane was used as an internal standard (0 ppm). CD spectra were recorded on a JASCO J-820 spectropolarimeter at 295 K. Mass spectra (ESI-TOF, positive mode) were recorded on an Applied Biosystems QStar Pulsar *i* spectrometer.

### Silylation of ethyl lactate (Fig. 5)

Under nitrogen atmosphere, *tert*-butyldimethylchlorosilane (10.0 g, 66.3 mmol) was added to a solution of (*S*)-ethyl lactate (7.2 mL, 63 mmol) and imidazole (5.15 g, 75.6 mmol) in dry dichloromethane (40 mL). The mixture was stirred for 2 h at room temperature. After addition of water, the mixture was extracted with dichloromethane. The combined organic layer was dried over anhydrous sodium sulfate, filtered, and concentrated to dryness. The crude oily product was purified by column chromatography (silica gel, ethyl acetate/hexane, 2:100) to give ethyl (*S*)-2-(*tert*-butyldimethylsilyloxy)propanoate (**E1** ^48^) (15.6 g, quant.) as colorless oil, ^1^H NMR (400 MHz, CDCl_3_) *δ* 0.07 (s, 3 H), 0.10 (s, 3 H), 0.91 (s, 9 H), 1.28 (t, *J* = 7.1 Hz, 3 H), 1.39 (d, *J* = 6.8 Hz, 3 H), 4.14–4.21 (m, 2 H), 4.31 (q, *J* = 6.8 Hz, 1 H).

**Figure 5:**
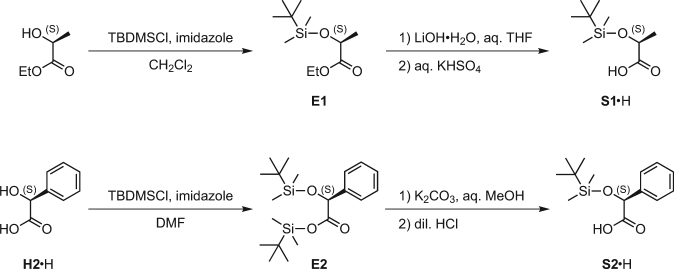
Synthetic schemes for **S1·**H and **S2·**H.

### Preparation of a stock solution of (*S*)-2-(*tert*-butyldimethylsilyloxy)propanoic acid (**S1**·H; Fig. 5)

An aqueous solution of lithium hydroxide monohydrate (49.3 mg, 1.17 mmol in 4 mL of water) was added dropwise to a solution of ester **E1** (119 mg, 0.510 mmol) in THF (4 mL) at 0 °C. The mixture was stirred for 4 h at room temperature and then concentrated. The solution was acidified to pH 4–5 with aqueous KHSO_4_ solution (1 M) and extracted with chloroform. The combined organic layer was dried over anhydrous sodium sulfate and filtered. The product **S1**·H^48^ was stored as chloroform solution, because **S1**·H gradually decomposes without solvent. ^1^H NMR (400 MHz, CDCl_3_) *δ* 0.15 (s, 6 H), 0.94 (s, 9 H), 1.46 (d, *J* = 7.0 Hz, 3 H), 4.36 (q, *J* = 7.0 Hz, 1 H) (Supplementary Fig. S1).

### Silylation of mandelic acid (**H2**·H; Fig. 5)

Under nitrogen atmosphere, *tert*-butyldimethylchlorosilane (307 mg, 2.04 mmol) was added to a solution of (*S*)-mandelic acid (**H2**·H, 96.0 mg, 0.631 mmol) and imidazole (190 mg, 2.79 mmol) in dry DMF (2 mL) at 0 °C. The mixture was stirred for 32 h at room temperature. After addition of water, the mixture was extracted with diethyl ether. The combined organic layer was dried over anhydrous sodium sulfate, filtered, and concentrated. The crude oily product was purified by column chromatography (silica gel, ethyl acetate/hexane, 3:7) to give ethyl (*S*)-2-(*tert*-butyldimethylsilyloxy)-2-phenylacetate (**E2** ^49^) (230 mg, 0.605 mmol, 95%) as pale yellow oil, ^1^H NMR (400 MHz, CDCl_3_) *δ* 0.01 (s, 3 H), 0.11 (s, 3 H), 0.14 (s, 3 H), 0.19 (s, 3 H), 0.82 (s, 9 H), 0.91 (s, 9 H), 5.14 (s, 1 H), 7.26–7.33 (m, 3 H), 7.44–7.47 (m, 2 H).

### Preparation of a stock solution of (*S*)-2-(*tert*-butyldimethylsilyloxy)-2-phenylacetic acid (**S2**·H; Fig. 5)

A solution of potassium carbonate in 50% aqueous methanol (1 M, 30 mL) containing ester **E2** (116 mg, 0.305 mmol) was heated to reflux for 1 h. After cooling to room temperature, the solution was concentrated. The residue was acidified to pH 4–5 with diluted hydrochloric acid (0.5 M) and the solution was extracted with chloroform. The combined organic layer was dried over anhydrous sodium sulfate and filtered. The product **S2**·H^49^ was stored as chloroform solution, because **S2**·H gradually decomposes without solvent. ^1^H NMR (400 MHz, CDCl_3_) *δ* –0.02 (s, 3 H), 0.13 (s, 3 H), 0.94 (s, 9 H), 5.20 (s, 1 H), 7.34–7.43 (m, 5 H) (Supplementary Fig. S2).

### Helicity inversion by F^–^ addition

A chloroform solution of the siloxycarboxylic acids (**S1**·H or **S2**·H, 3 equiv) was added to an acetonitrile solution of LZn_3_La^40^ in the presence of DABCO (3 equiv). After 5 min, an acetonitrile solution of tetrabutylammonium fluoride (3 or 4 equiv) was added to the solution and the time course of the CD spectral changes was investigated. The solvent ratio of the solution was adjusted to be acetonitrile/chloroform = 9:1.

### Data availability

Data supporting the findings of this study are available within the article (and its Supplementary Information files) and from the corresponding author on reasonable request.

## Electronic supplementary material

Supplementary Information
