# Supplementary material for: Response speed control of helicity inversion based on a “regulatory enzyme”-like strategy
Source: Sci Rep. 2018 Jan 9;8:137. doi: 10.1038/s41598-017-16503-1 (PMC5760571; doi:10.1038/s41598-017-16503-1)
Supplement: Supplementary file 1 — Supplementary Information [file 41598_2017_16503_MOESM1_ESM.doc]

**Response speed control of helicity inversion based on a “regulatory enzyme”-like strategy**

Shiho Sairenji,^†^ Shigehisa Akine,^‡^* and Tatsuya Nabeshima^†^*

**Supplementary Information**

**Affiliations and contact information**

† Faculty of Pure and Applied Sciences, University of Tsukuba, 1-1-1 Tennodai, Tsukuba, Ibaraki 305-8571, Japan

Phone/Fax: +81-29-853-4507

E-mail: nabesima@chem.tsukuba.ac.jp

‡ Graduate School of Natural Science and Technology / Nano Life Science Institute (WPI-NanoLSI), Kanazawa University, Kakuma-machi, Kanazawa 920-1192, Japan

Phone: +81-76-264-5701; Fax: +81-76-264-5742

E-mail: akine@se.kanazawa-u.ac.jp


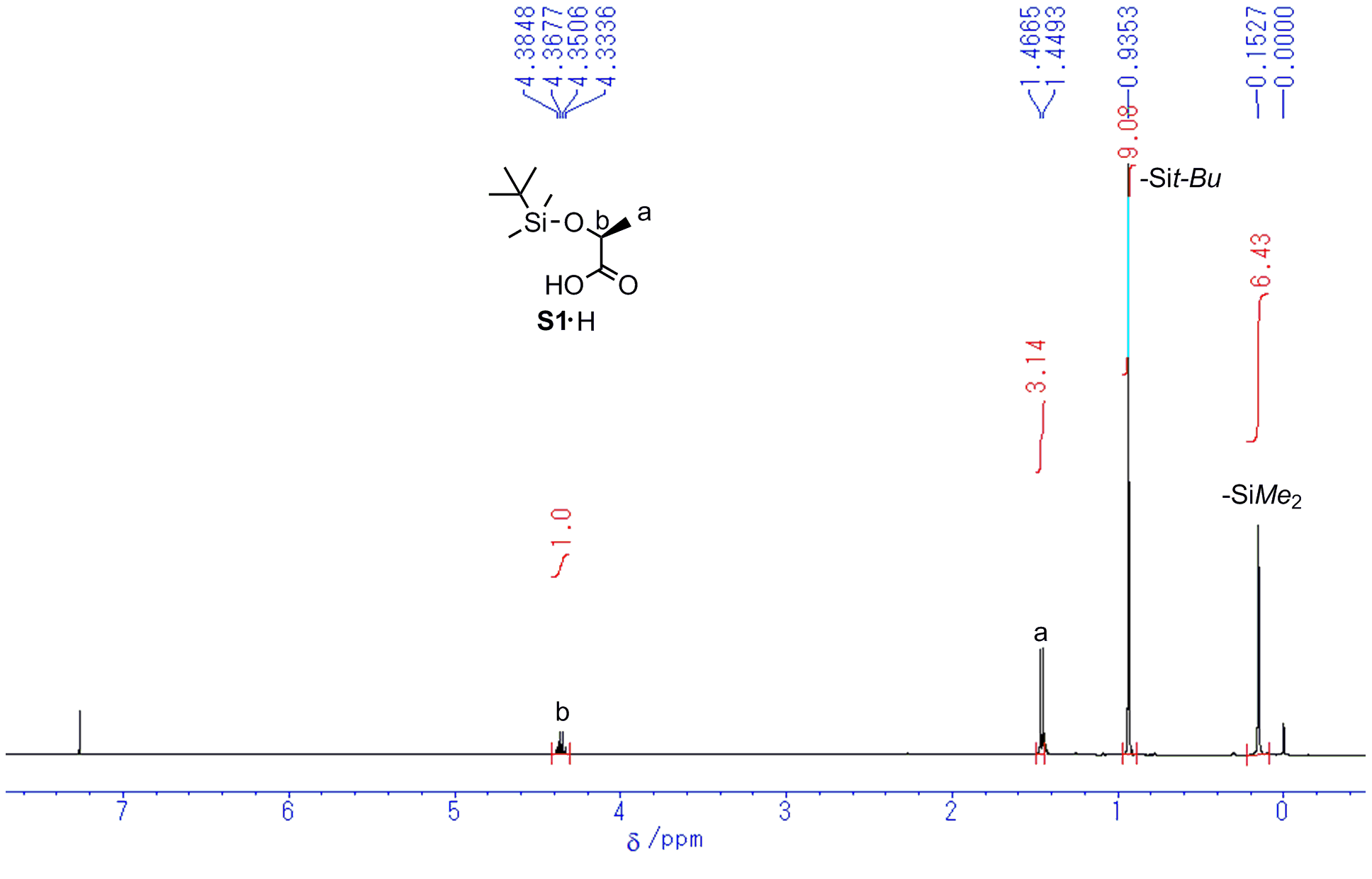


**Supplementary Fig. S1.** ^1^H NMR spectrum of **S1**·H (400 MHz, CDCl_3_).


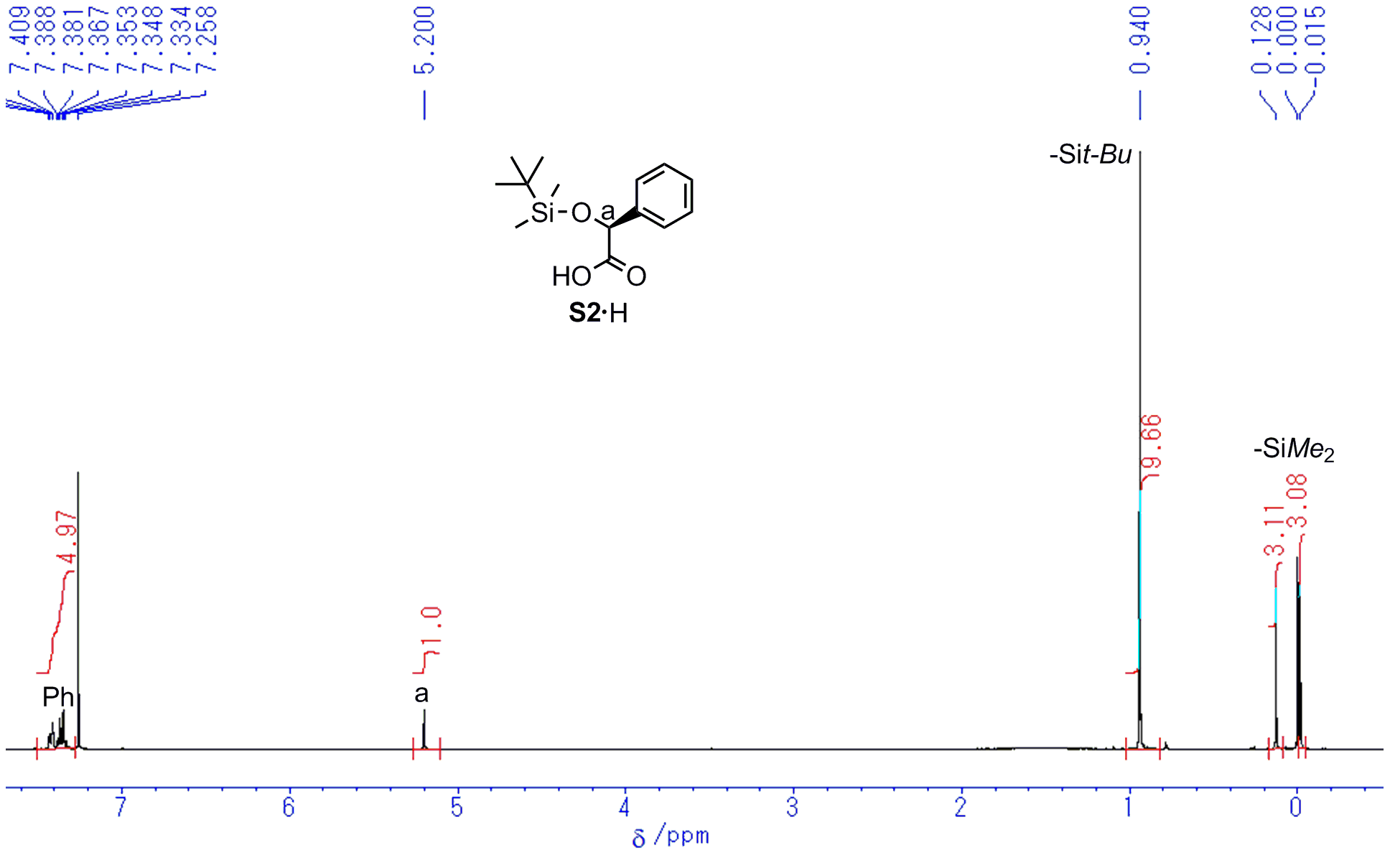


**Supplementary Fig. S2.** ^1^H NMR spectrum of **S2**·H (400 MHz, CDCl_3_).


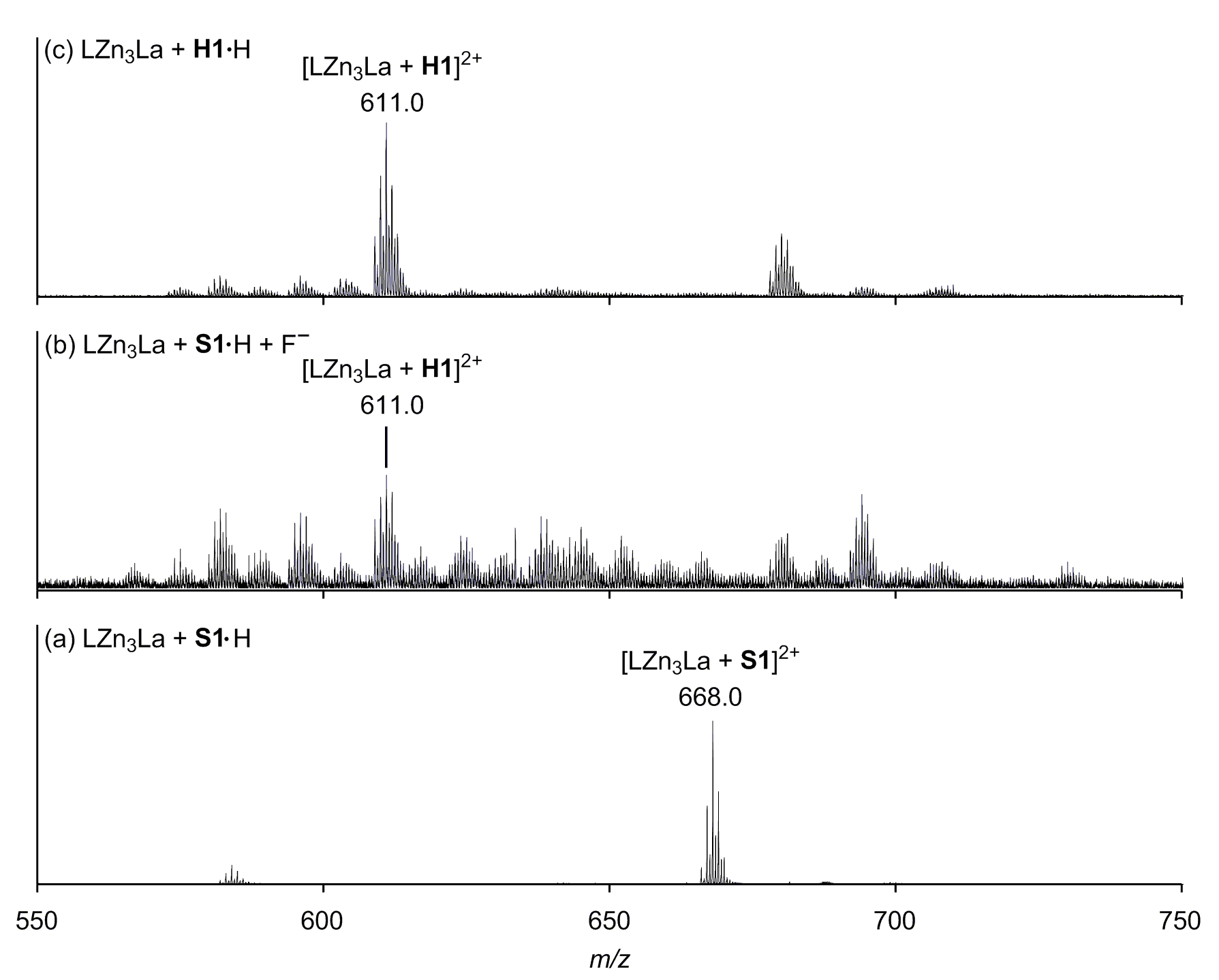


**Supplementary Fig. S3.** ESI mass spectra of LZn_3_La in the presence of carboxylic acids and DABCO. (a) **S1**·H, (b) **S1**·H after the addition of tetrabutylammonium fluoride, and (c) **H1**·H.


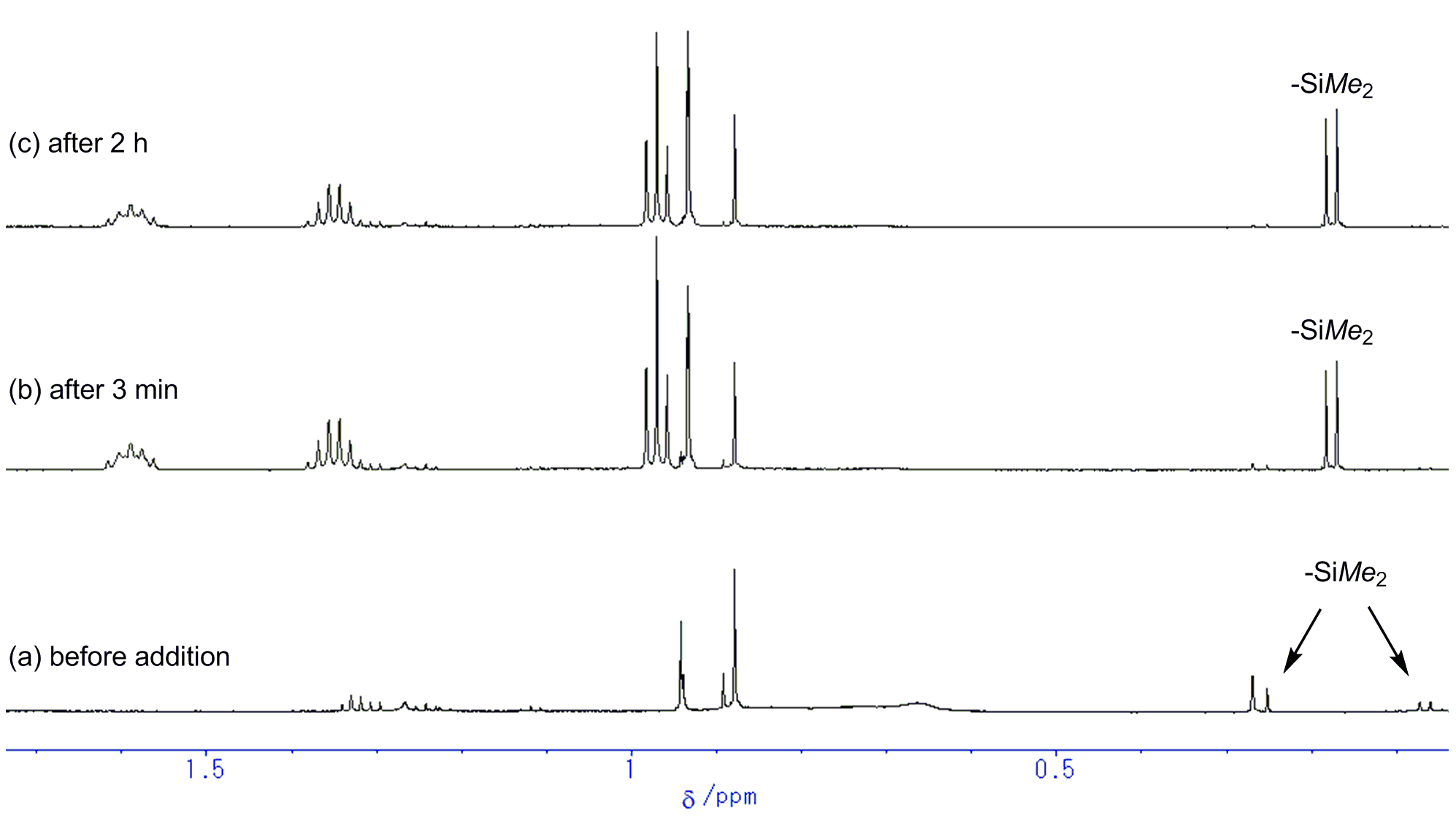


**Supplementary Fig. S4.** ^1^H NMR spectral changes of LZn_3_La in the presence of **S1**·H (3 equiv) and DABCO (3 equiv) (600 MHz, 0.20 mM, CD_3_CN/CDCl_3_, 9:1). (a) before F^–^ addition, (b) 3 min after F^–^ addition, and (c) 2 h after F^–^ addition.


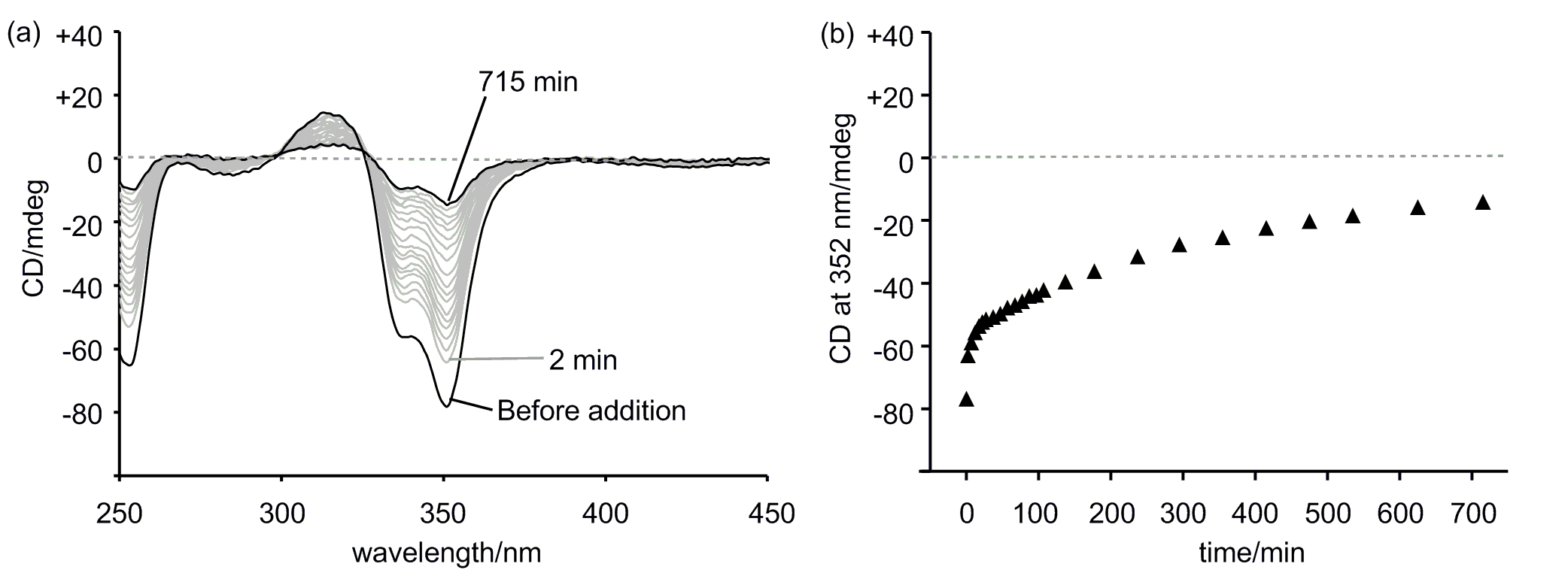


**Supplementary Fig. S5.** CD spectral observation of helicity inversion triggered by F^–^ addition. (a) CD spectral changes and of LZn_3_La (0.20 mM, acetonitrile/chloroform, 9:1, 295 K) in the presence of **S2**·H (3 equiv) and DABCO (3 equiv) after the addition of tetrabutylammonium fluoride (3 equiv). (b) Time course of the CD intensity changes (352 nm).


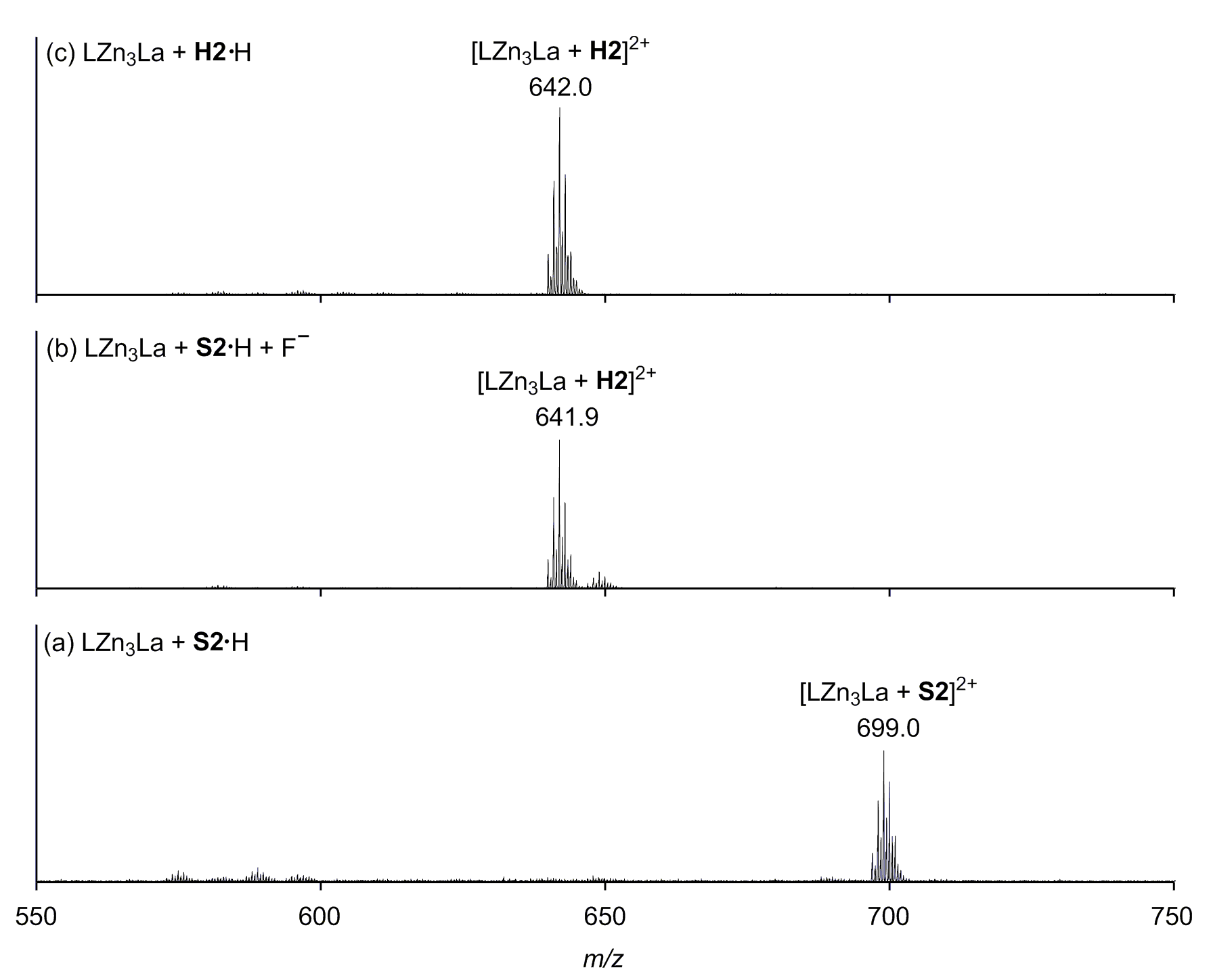


**Supplementary Fig. S6.** ESI mass spectra of LZn_3_La in the presence of carboxylic acids and DABCO. (a) **S2**·H, (b) **S2**·H after the addition of tetrabutylammonium fluoride, and (c) **H2**·H.


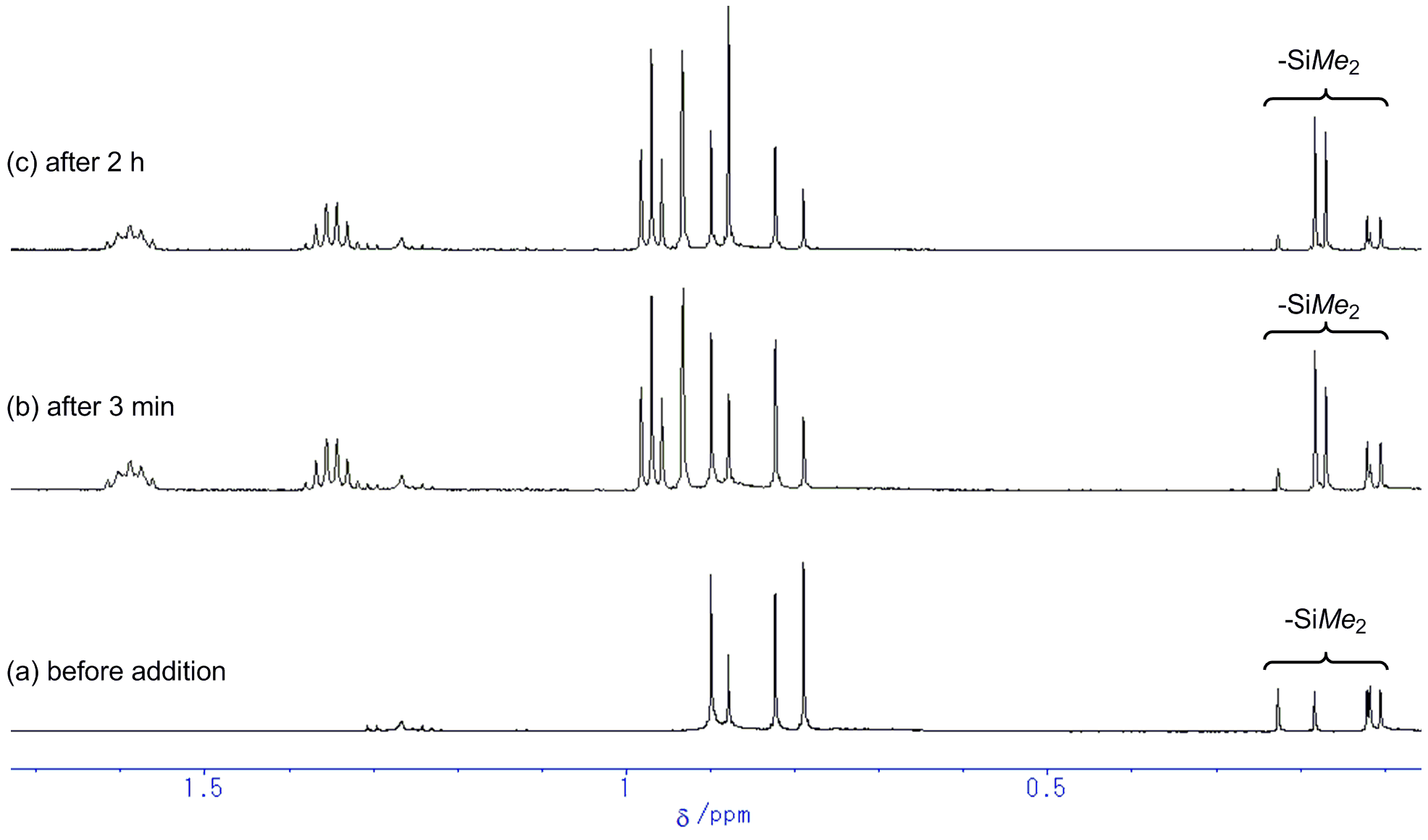


**Supplementary Fig. S7.** ^1^H NMR spectral changes of LZn_3_La in the presence of **S2**·H (3 equiv) and DABCO (3 equiv) (600 MHz, 0.20 mM, CD_3_CN/CDCl_3_, 9:1). (a) before F^–^ addition, (b) 3 min after F^–^ addition, and (c) 2 h after F^–^ addition.
